# Intestinal ion regulation exhibits a daily rhythm in *Gymnocypris przewalskii* exposed to high saline and alkaline water

**DOI:** 10.1038/s41598-021-04472-5

**Published:** 2022-01-17

**Authors:** Fei Wang, Lin Zhu, Yuxing Wei, Pengcheng Gao, Yimeng Liu, Kai Zhou, Zhen Sun, Qifang Lai, Zongli Yao

**Affiliations:** 1 Engineering Research Center for Saline-alkaline Fisheries, Sino-US Joint Laboratory of Aquatic Animal Physiology, East China Sea Fisheries Research Institute, Chinese Academy of Fishery Sciences, Shanghai, China; 2 National Demonstration Center for Experimental Fisheries Science Education, Shanghai Ocean University, Shanghai, China

**Keywords:** Ecophysiology, Ecophysiology, Ocean sciences

## Abstract

Naked carp (*Gymnocypris przewalskii*), endemic to the saline-alkaline Lake Qinghai, have the capacity to tolerate combinations of high salinity and alkalinity, but migrate to spawn in freshwater rivers each year. In this study, we measured the drinking rate over a 24 h period for naked carp exposed to saline-alkaline lake waters with salinities of 15 (L15) and 17 (L17). We also assessed the daily feed intakes of naked carp exposed to L15 and fresh water (FW). Additionally, we studied the daily expression of acid–base regulation and osmoregulation related genes and proteins in the intestine of naked carp exposed to saline-alkaline lake waters. Our results revealed that the drinking rate at night was significantly higher than in daytime when exposed to either L15 or L17, while feed intakes in daytime were significantly higher than at night. The relative expression of Na^+^/K^+^-ATPase α (NKA-α), solute carrier family members 26A6 (SLC26A6) and 4A4 (SLC4A4) in the intestine of naked carp exposed to L17 at night was higher than in daytime. Specifically, NKA-α mRNA expression at 4:00 was 7.22-fold and 5.63-fold higher than that at 10:00 and 16:00, respectively, and the expression at 22:00 was 11.29-fold and 8.80-fold higher than that at 10:00 and 16:00, respectively. Similarly, SLC26A6 mRNA expression was greatest at 22:00, exceeding that observed at 4:00, 10:00 and 16:00 by 3.59, 4.44 and 11.14-fold, respectively. Finally, the expression of NKA-α and SLC26A6 protein at the single cell level was also higher at night than during the day, which was 1.65-fold and 1.37-fold higher at 22:00 respectively compared to 16:00. Overall, the present findings revealed that naked carp drinks at night and feeds during the day, demonstrating that intestinal ion regulation exhibits a daily rhythm when exposed to high saline and alkaline lake water.

## Introduction

*Gymnocypris przewalskii*, also known as naked carp or the scale-less carp, is endemic to the extreme environment of Lake Qinghai^1^. Lake Qinghai has a low concentration of dissolved oxygen (4.16–6.08 mg L^−1^), strong alkalinity (carbonate alkalinity approximately 29 mmol L^−1^, pH 9.1–9.5), and a high salinity (13–15 ppt)^2^. Due to evaporation and agricultural irrigation, the salinity and alkalinity levels of Lake Qinghai are increasing by 7 and 0.5% per year, respectively^3^. Although historically rich in resources, the naked carp population decreased significantly because of climate change, overfishing and destruction of spawning habitat^4^.

Teleost fish living in seawater drink surrounding water to avoid dehydration. This plays an important role in their osmoregulation and presents the challenge of salts excretion and water absorption^5^. While not full strength seawater, the salinity of Lake Qinghai is nevertheless hyperosmotic to naked carp, thus necessitating the need for these fish to drink in order to effectively osmoregulate^6^.

The osmoregulatory mechanism of fish inhabiting such saline-alkaline water is unknown. Bergman et al.^7^ described the intestinal structure and physiology of the Lake Magadi tilapia (*Alcolapia grahami*) when feeding and drinking in highly alkaline water and found that they differ from other freshwater and marine teleosts. The esophagus, stomach and intestine form an unusual configuration ‘T’ that allows for the selective shunting of ingested food and water. The pyloric sphincter is open when the fish is drinking and closed when the fish is eating. This unique configuration allows water to almost completely bypass the stomach and enter directly into the intestine^7^. However, the naked carp has no stomach and thus cannot physically partition intestinal water absorption from food digestion. Moreover, additional research has shown that the digestive and drinking behavior of fish in general cannot be performed at the same time^8^. This led us to question, whether a stomachless fish, such as naked carp might partition these functions temporally rather than physically.

The mechanisms of daily rhythm influenced by the periodic changes of environmental factors such as temperature and illumination, and have a coordinated effect on life activities such as physiology and behavior^9,10^. The regulation mechanism of fish feeding rhythm integrates external signals with peripheral and central nervous system by brain-gut axis^11^. It was recently discovered that the intestinal rhythm is mainly related to light and food^12^. Feeding behavior of fish is limited by sensory ability. Fish, those ingest rely on vision, usually feed during the day. Fish with poor visual ability are more active at night^13^. In addition, the daily rhythm of some fish appears to be more flexible than that of higher vertebrates^14^. For example, in natural environments, rainbow trout (*Oncorhynchus mykiss*) swims to the bottom to feed during the day and rises to the surface for locomotor activity at night^15^, while in Nile tilapia (*Oreochromis niloticus*) the situation is reversed^14^. In addition, goldfish (*Carassius auratus*)^16^, Atlantic salmon (*Salmo salar* L.)^17^, silver carp (*Hypophthalmichthys molitrix*), bighead carp (*Hypophthalmichthys nobilis*)^18^ have obvious daytime feeding behaviors, while mackerels^19^ feed more intensely at night. On the other hand, European sea bass (*Dicentrarchus labrax* L.) exhibit continuous feeding characteristics over both diurnal and nocturnal scales^20^. While zebrafish (*Danio rerio*) developed a food anticipatory activity, displayed locomotor activity mostly during the daytime^21^.

The intestine has the function of both digestion and osmoregulation^22^. The food intake of fish is not only affected by external factors such as temperature and photoperiod, but also by internal factors such as gastrointestinal tract fullness and gastrointestinal tract emptying rate^23^. And the intestine digestion showed obvious daily rhythm. For example, the trypsin and chymotrypsin activities in foregut, midgut and hindgut of Nile tilapia showed significant daily rhythms. The enzyme activity in daytime was higher than that in night^24^. Moreover, the osmoregulatory role of the intestine is linked to acid–base balance in marine and euryhaline teleosts and its ability to effectively absorb fluid is essential to compensate for water loss in hypertonic environments^5,25^. Previous results have shown that Na^+^/K^+^-ATPase (NKA) expression and activity increase following transition from freshwater to seawater^26^. The majority of teleost fish regulate acid–base balance by adjusting plasma HCO_3_^−^ levels through the differential regulation of H^+^ and HCO_3_^−^ transport in exchange for Na^+^ and Cl^−^, respectively^27^, via exchangers such as solute carrier family members 26A6 (SLC26A6), solute carrier family members 4A4 (SLC4A4). However, an apparent exception is the Lake Magadi tilapia which showed more than 70% of intestinal flux occurred via Na^+^ and base co-transport, and less than 30% by Na^+^ and Cl^−^ co-transport^7^.

In the end, the question of how stomach less fish living in a hypersaline environment, such as naked carp, are able to sufficiently balance water absorption and food digestion without a means of physical partitioning within the gut. We hypothesized that naked carp living in a high saline-alkaline water environment partition these functions temporally such that they drink at night and feed during the day. Studies on *G. przewalskii* have shown that the mRNA expression of SLC26 and SLC4 gene families were up-regulated in the intestine when exposed to different saline-alkaline waters. This was particularly evident under conditions of high salinity and alkalinity, where the up-regulation of expression was clear, indicating that under these conditions naked carp secrete and excrete the accumulated HCO_3_^−^ in vivo through the intestine Cl^−^/HCO_3_^−^ exchanger and Na^+^-HCO_3_^−^ co-transporter^6^, respectively. Furthermore, a decrease in cytosolic carbonic anhydrase c expression in these conditions has been shown, likely reflecting a compensatory response to a respiratory alkalosis and as a means for aiding in osmoregulation during the transition from a freshwater river to a saline-alkaline lake^3^. With this background in mind, the focus of this study was to investigate the potential effect of a daily rhythm on osmoregulation, acid–base balance and feeding in naked carp living in the saline-alkaline water of Lake Qinghai. In this study, naked carp were placed in saline-alkaline lake waters with salinities of 15(L15) and 17(L17). We measured the drinking rate over a 24 h period in both treatments. Additionally, we measured the daily feed intakes of naked carp exposed to saline-alkaline lake water (L15) and fresh water (FW).We also studied the daily expression of genes and proteins, which related to acid–base regulation and osmoregulation, in the intestine of naked carp exposed to saline-alkaline lake waters.

## Results

### Drinking rate

**Figure 1:**
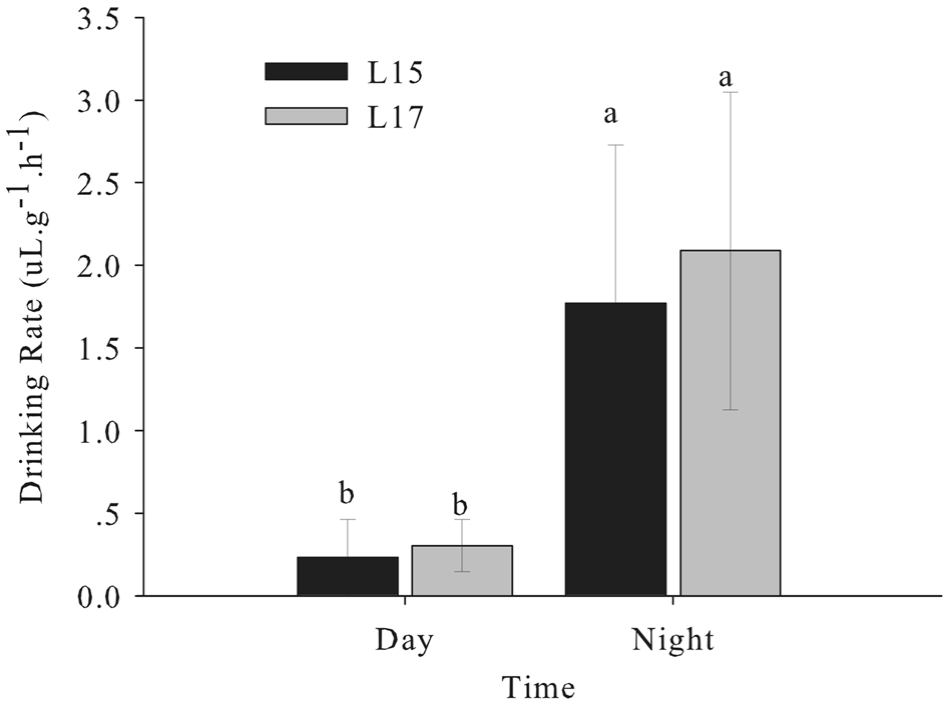
Drinking rates of *Gymnocypris przewalskii* exposed to saline-alkaline lake water (L15 and L17) at different times (Day 10:00–16:00, Night 22:00–4:00) (n = 9 per treatment). Light–dark cycle was 14:10-h light:dark (5:00–19:00 with light intensity of 600–1000 lx;19:00–5:00 with light intensity of 0 lx); L15 and L17 refers to saline-alkaline lake water with a salinity of 15 or 17. Lowercase indicates significantly difference among time points in each treatment (*P* < 0.05).

Drinking rate of naked carp exposed to L15 and L17 were assessed (Fig. 1). In each treatment, drinking rate of naked carp at night (22:00–4:00) were significantly higher than that in the daytime (10:00–16:00) (*P* < 0.05). In the L15 treatment, the drinking rate at night was 1.77 uL g^−1^ h^−1^, which was significantly higher (*p* < 0.05) than that in the daytime. In the L17 treatment, the drinking rate at night was 2.09 uL g^−1^ h^−1^, which was significantly higher (*p* < 0.05) than that in the daytime.

### Daily feed intake

**Figure 2:**
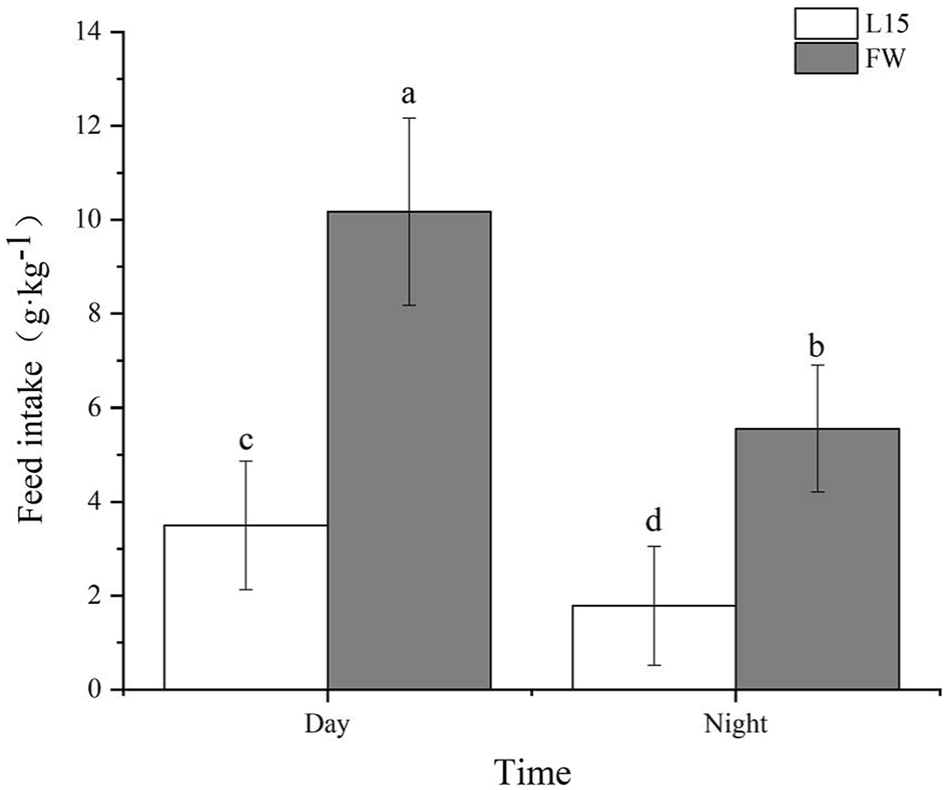
Daily feed intakes of *Gymnocypris przewalskii* exposed to saline-alkaline lake water (L15) and fresh water (FW) at different times (Day 5:00–19:00, Night 19:00–5:00) (n = 3 per treatment). Light–dark cycle was 14:10-h light:dark (5:00–19:00 with light intensity of 600–1000 lx;19:00–5:00 with light intensity of 0 lx); L15 refers to saline-alkaline lake water with a salinity of 15; Lowercase indicates significantly difference between phases in each treatment (*P* < 0.05).

Daily feed intakes of naked carp exposed to L15 and FW were assessed (Fig. 2). In each treatment, daily feed intakes in the daytime were significantly higher than those at night. In the L15, the daily feed intake in the daytime was 3.50 g kg^−1^ which was significantly higher (*P* < 0.05) than that at night. In FW, the daily feed intake in the daytime was 10.17 g kg^−1^ which was significantly higher (*P* < 0.05) than that at night.

### Gene expression in response to saline-alkaline exposure

**Figure 3:**
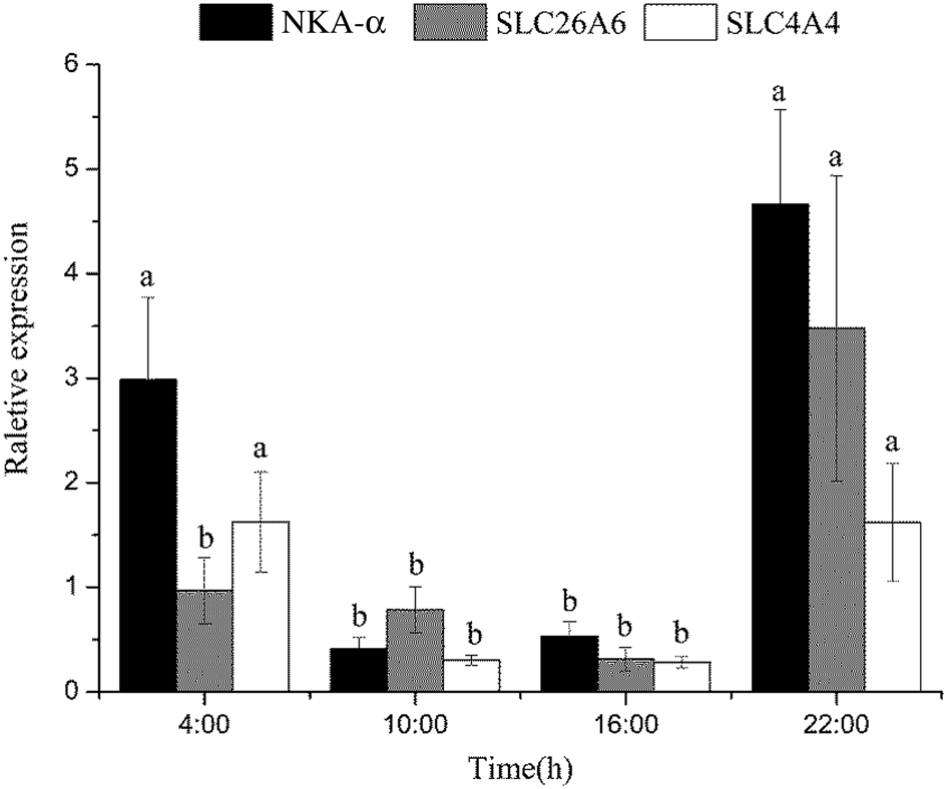
NKA-α, SLC26A6, SLC4A4 mRNA expression levels in the mid-intestine of *Gymnocypris przewalskii* at different time points (4:00, 10:00, 16:00 and 22:00) exposed to saline-alkaline lake water with a salinity of 17. Light–dark cycle was 14:10-h light:dark (5:00–19:00 with light intensity of 600–1000 lx;19:00–5:00 with light intensity of 0 lx); Lowercase indicates significantly difference among time points in each treatment (*P* < 0.05).

Expression of NKA-α, SLC26A6 and SLC4A4 in intestine of naked carp exposed to L17 were investigated (Fig. 3). In L17, NKA-α mRNA expression at 4:00 and 22:00 was significantly (*P* < 0.05) higher than other time points. The expression at 4:00 was 7.22-fold and 5.63-fold that of 10:00 and 16:00, respectively, and the expression at 22:00 was 11.29-fold and 8.80-fold that of 10:00 and 16:00, respectively; SLC26A6 mRNA expression at 22:00 was significantly (*P* < 0.05) higher than the other time points, which was 3.59-fold of 4:00, 4.44-fold of 10:00 and 11.14-fold of 16:00. The expression of SLC4A4 mRNA at 4:00 and 22:00 was significantly (*P* < 0.05) higher than at 10:00 and 16: 00. The expression at 4:00 was 5.34-fold of 10:00 and 5.75-fold of 16:00, whereas the expression at 22:00 was 5.32-fold of 10:00 and 5.73-fold of 16:00.

### Intestinal single cell positivity rates for NKA-α and SLC26A6

**Figure 4:**
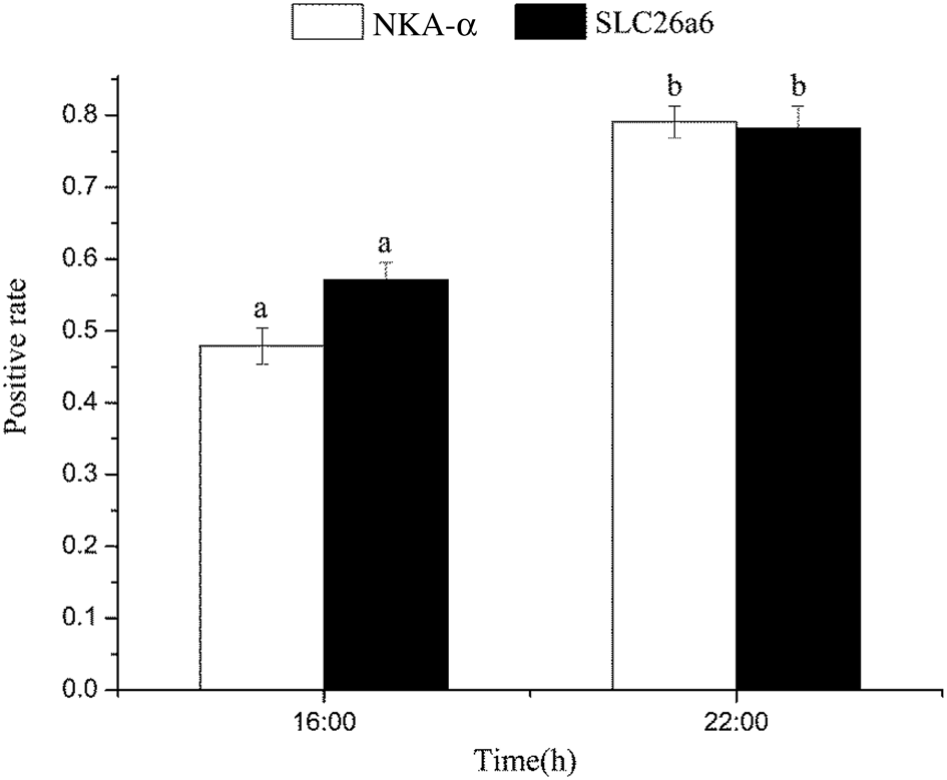
NKA-α, SLC26A6 positivity rate in the mid-intestine of *Gymnocypris przewalskii* at different time points exposed to saline-alkaline lake water with a salinity of 17 (L17). Light–dark cycle was 14:10-h light:dark (5:00–19:00 with light intensity of 600–1000 lx;19:00–5:00 with light intensity of 0 lx); Lowercase indicates significant difference among time points in each treatment (*P* < 0.05); Positive rate = protein staining/nuclear staining. Image J was used for measurement, analysis and calculation).

**Figure 5:**
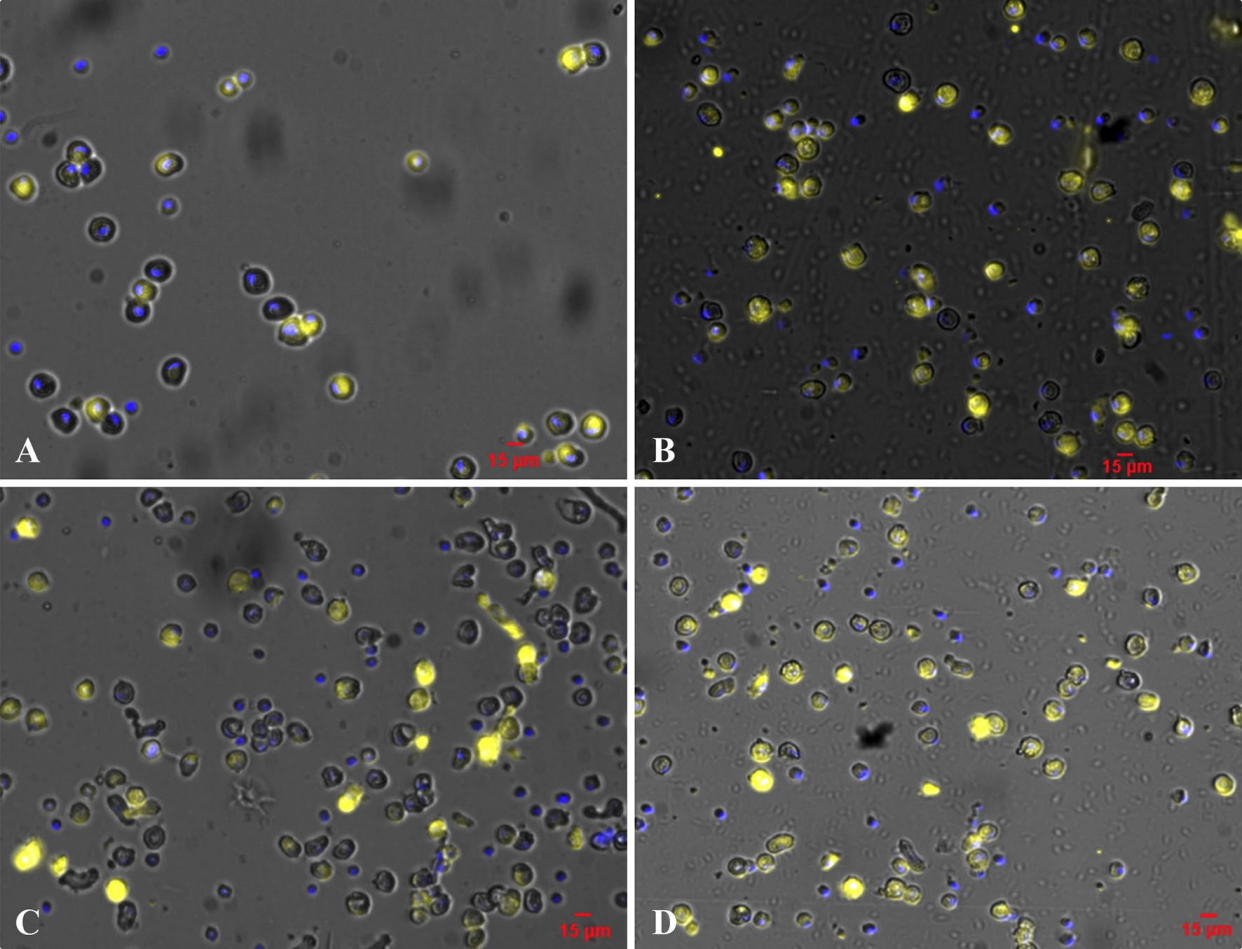
Example of single cell positive expression. Nuclear staining is shown in blue and NKA-α and SLC26a6 staining is shown in yellow. (**A**) The expression of NKA-α at 16:00. (**B**) The expression of NKA-α at 22:00. (**C**) The expression of SLC26A6 at 16:00. (**D**) The expression of SLC26A6 at 22:00.

Western blot analysis for NKA-α and SLC26A6 revealed distinct bands of ~ 113 kDa and ~ 50 kDa respectively (Fig. S1). Single cell positivity rates for NKA-α and SLC26A6 in naked carp in response to L17 acclimation from fresh water were investigated in mid-intestine. In L17, there was significant differences at each time point (*P* < 0.05) (Fig. 4). The expression of NKA-α and SLC26A6 at 22:00 was 1.65-fold and 1.37-fold of 16:00, respectively. Figure 5 shows the protein staining results of NKA-α and SLC26A6 at 16:00 and 22:00, respectively.

## Discussion

Teleosts living within hyperosmotic environments are faced with the constant challenges of diffusive water loss and ion gain^25^. Thus, to maintain osmoregulatory homeostasis in these environments^28^, teleosts must actively drink the surrounding water, absorb salts and water across the gut and then actively secrete the added salt load across the gills^29^. The naked carp migrates annually between freshwater rivers and Lake Qinghai as part of their life history strategy^30^. Thus, migrating to lake water demands a transition in osmoregulatory strategy as the fish moves from a hypoosmotic environment to a hyperosmotic environment. Considering that the naked carp lacks a stomach for physically partitioning digestion and absorption, we hypothesized that these fish might partition these functions temporally when moving into the hyperosmotic lake water. To address this, we analyzed the daily changes in feeding, drinking and expression/activity of key proteins involved in digestion and osmoregulation of naked carp in saline-alkaline environments. Our results first time revealed that naked carp exhibit a daily rhythm whereby drinking is partitioned more to evening hours and feeding is partitioned to a greater extent during the day. This was supported by the observations that the drinking rate and expression of key osmoregulatory genes of naked carp at night were significantly higher than that observed during the day, while the feed intake was significantly lower at night than during the day.

Transporters involved in the regulation of intestinal epithelial ions mainly include NKA, Cl^−^/HCO_3_^−^ exchangers, Na^+^-HCO_3_^−^ co-transporter and others. When teleost fish are transferred to seawater, osmoregulation is maintained by increasing the expression of NKA mRNA and its activity in intestinal epithelial cells^31,32^. The SLC4 and SLC26 gene families are the main proteins involved in HCO_3_^−^ transport in organisms, and play an important role in maintaining the pH and ion balance of body fluids^33,34^. When naked carp were transferred directly from fresh water to saline-alkaline water, the expression of NKA increased in the basolateral membrane of the intestine likely as a response to increase salt uptake and maintain the osmotic pressure gradient for water absorption. Yao et al.^3^ found that the naked carp transferred from river water to lake water resulted in a respiratory alkalosis and ion rise in the blood. The expression of SLC26A6 and SLC4A4, on the other hand, likely increased as a compensatory measure to aid in the secretion and excretion of accumulated bicarbonate concentrations^6^. Perhaps most notably, the drinking rate of naked carp was significantly higher at night than that during the day which aligned with the heightened expression of NKA-α, SLC26A6 and SLC4A4, further supporting that osmoregulation and acid–base balance appear to be partitioned to a greater extent during this period of the daily cycle.

Fish usually exhibit certain feeding rhythms that can be connected with external environment and internal factors. Generally, feeding rhythms represent the evolutionary trade-off between food availability, metabolic demand and predator risk^35^. During the experiment, the mode of self-feeding was closer to the survival conditions of natural environment than the mode of artificial feeding by schedules. The use of self-feeders can not only promote growth, improve feed conversion and reduce feed waste, but also find some behavioral characteristics related to self-feeding activities^36^. The study found that self-feeding intake of naked carp was higher in the daytime, indicating increased feeding behavior during the day. Naked carp triggered the self-feeder when they need to ingest feed. The daily feed intakes of L15 and FW in daytime was 3.50 g kg^−1^ and 10.17 g kg^−1^, respectively, significantly higher than that of 1.79 g kg^−1^ and 5.55 g kg^−1^ at night. This indicated that high feed intake in daytime could depend on surrounding lights, since visual feeder usually feed during the day^13^. In addition, the intestinal tract of fish can improve digestive physiological activities through rhythmic digestive enzyme activities, and then affect feeding activities^37^. Daily rhythms have evolved from long-term adaptation of life to daily changes of environmental factors on the Earth^9,38^. Studies represented by zebrafish have shown that fish exhibit distinct daily rhythms in physiology and behavior^21^, which include feeding rhythms.

In conclusion, naked carp appear to cope with the conflicting demands of feeding/digestion and drinking/osmoregulation by alternating these functions within a daily rhythm, whereby the former is largely achieved during the day and the latter by night. This was supported by the fact that the drinking rate of naked carp at night was significantly higher than that during the day while the feed intakes exhibited the opposite pattern. Moreover the relative expression of key osmoregulatory and acid–base proteins (i.e., NKA, SLC26A6 and SLC4A4) paralleled the daily changes in drinking rate. Given the high salinity/alkalinity environment of naked carp, we predict that the daily rhythm may therefore play an important role in osmoregulation and ion regulation of this fish.

## Methods

### Experimental animals

*Gymnocypris przewalskii* used in this study were obtained from the Rescue Center for Naked Carp of Lake Qinghai in Xining. Only healthy fish without visible body damage were used. Wet mass and body length were recorded before the fish were sampled. All fish were collected under permits issued by local and national authorities, and experimental procedures were in accordance with national animal care regulations. Experimental waters were prepared daily, and water qualities were measured before each experiment. The water temperature and salinity were measured using an YSI6600 multiprobe sensor (YSI Incorporated, Ohio, USA), and the carbonate alkalinity was determined by titration^39^. All fish (average body weight: 33.21 ± 2.74 g; average body length:14.81 ± 0.35 cm) were held in an indoor RAS system at a density of approximately 6.5 kg m^−3^. The holding and experimental water were filtered tap water (Canature/AC/KDF150-1–300) (salinity 0.16, pH 7.56, carbonate alkalinity 2.7 mmol L^−1^, temperature 17.1 ± 0.61 °C). Fish were fed daily with commercial feed. Fish husbandry and experimental procedures were approved by the Second Scientific Research Ethics Committee of East China Sea Fisheries Research Institute.

### Experimental design

Fish were placed on a 14:10-h light:dark (5:00–19:00 with light intensity of 600–1000 lx; 19:00–5:00 with light intensity of 0 lx) photoperiod aquaculture system. To examine the effect of rhythm on osmoregulation and acid–base balance, this study measured four endpoints: drinking rate, self-feeding intake, mRNA expression and the single cell expression level of osmoregulation and acid–base regulation relevant proteins. Fish held in filtered tap water were transferred directly to saline-alkaline lake water with salinities of 15 (L15, salinity 14.83, pH 8.65, carbonate alkalinity 30.54 mmol L^−1^) and 17 (L17, salinity 16.80, pH 9.02, carbonate alkalinity 34.61 mmol L^−1^), which was prepared by adding the same ratio of NaCl, MgCl_2_.6H_2_O, Na_2_SO_4,_ CaCl_2_, KCl, NaHCO_3_ and Na_2_CO_3_ as in Qinghai Lake. The experimental period was 4–5 days.

### Drinking rate during high saline-alkaline transfer

Fish were placed on a 14:10-h light:dark (5:00–19:00 with light intensity of 600–1000 lx;19:00–5:00 with light intensity of 0 lx) photoperiod aquaculture system. In this experiment, feeding was stopped 48 h prior to the experiments. Fresh water fish were transferred directly to L15 and L17 PEG-4000 free water for up to 4 days. For drinking rate analysis, new tanks were prepared which contained 50L saline-alkaline lake water with salinity 15 or 17 and with PEG-4000 (final concentration was 1.00 g L^−1^) during the day (10:00–16:00) and night (4:00–22:00) on the fourth day respectively. Nine fish per treatment were individually transferred from PEG-4000 free water to each tank which contained 1.00 g L^−1^ PEG-4000 at 10:00 or 4:00. Water samples were collected at 15 min after the fish were transferred to each treatment group for the determination of PEG-4000 concentration. The fish were terminally anesthetized with MS-222 (0.40 g L^−1^) after 6 h. The intestines were then quickly dissected out from nine individual fish per treatment group and the intestinal fluid were collected and stored at 4 °C. All fish were weighed before sampling.

### Self-feeding intake during high saline-alkaline transfer

Fish were placed on a 14:10-h light:dark (5:00–19:00 with light intensity of 600–1000 lx;19:00–5:00 with light intensity of 0 lx) photoperiod aquaculture system. In this experiment, fish were kept in freshwater (FW) or acclimated to L15 for more than 15 days before the experiment started. Six RAS glass tanks (95 cm × 60 cm × 60 cm), which belong to two circulatory systems (3 tanks for FW and 3 for L15), were used for self-feeding experiment. Each tank had 15 individuals.

Before the experiment, fish were trained by a custom-made self-feeding system (Fig. S2). Trained fish triggered the self-feeder when they want to feed. In the self-feeding system, the photoelectric sensor converts the change of optical signal into the change of electrical signal, and the feeder release feed by recognizing level fluctuation.

During the formal experiment, we collected feed data at 5:00 and 19:00, which were the time points of the light and dark transition. Feed intakes of naked carp were calculated by weighing the feed quantities at two time points. The experiment lasted 5 days.

### mRNA expression of osmoregulation and acid–base regulation relevant proteins during high saline-alkaline transfer

Fish were placed on a 14:10-h light:dark (5:00–19:00 with light intensity of 600–1000 lx;19:00–5:00 with light intensity of 0 lx) photoperiod aquaculture system. In this experiment, feeding was stopped 48 h prior to the experiments. Fresh water fish were transferred directly to L17 for up to 4 days. There were 24 fish per tank in triplicate. At the fourth day, six fish per tank were individually removed and terminally anesthetized with MS-222 (0.40 g L^−1^) at 4:00, 10:00, 16:00 and 22:00, respectively. The mid-intestine was quickly dissected out from six individual fish at each time point. Mid-intestine tissues for mRNA expression analyses were immediately snap-frozen in liquid N_2_, and stored at − 80 °C until analysis.

### Single cell positive rate of osmoregulation and acid–base regulation relevant proteins

Fish were placed on a 14:10-h light:dark (5:00–19:00 with light intensity of 600–1000 lx;19:00–5:00 with light intensity of 0 lx) photoperiod aquaculture system. In this experiment, feeding was stopped 48 h prior to the experiments. To analyze the single cell positive rate of acid–base relevant proteins, a separate experiment was conducted. Fresh water fish were transferred directly to L17 for up to 4 days. There were 3 tanks (6 fish per tank) in this experimental group. At the fourth day, three fish per tank were individually removed and terminally anesthetized with MS-222 (0.40 g L^−1^) at 16:00 and 22:00, respectively. The mid-intestine was quickly dissected out from nine individual fish and immediately prepared for single-cell suspensions.

### Analytical techniques

#### Drinking rate analysis

The measurement of drinking rate was performed according to the study of Buxton et al.^40^. After weighing the collected intestinal fluid, it was centrifuged at 13,000g for 1 min, and 50 μL of the supernatant was taken, added dropwise to 350 μL of 72% pre-cooled (4 °C) acetone, and vortexed to mix. Samples were then centrifuged at 2000g for 10 min at 4 °C, the supernatant filtered with 0.45 μm filter paper, followed by addition of 100 μL of filtrate to 175 μL 25 mg L^−1^ gum arabic and vortexed to mix. Finally, 200 μL of TCA-CaCl_2_ (trichloroacetic acid-calcium chloride, 30% and 5% by mass) was added to the mixture and the reaction allowed to proceed at room temperature for 20 min. An Epoch microplate (Bio Tek) spectrophotometry unit was used to measure the absorbance at 650 nm. The remaining solution was weighed again after drying at 60 °C for 48 h, and the volume of intestinal fluid was determined (quantity of collected intestinal fluid-mass after drying). The same method as above was used to process the standard solution. Solute concentrations for standard curve were prepared as 0.00 g L^−1^, 0.10 g L^−1^, 0.20 g L^−1^, 0.40 g L^−1^, 0.60 g/L^−1^, 0.80 g L^–1^, 1 g L^−1^, and 2 g L^−1^ PEG-4000. The PEG-4000 concentration of intestinal fluid was calculated based on the standard curve. Drinking rate (μLg^-1^h^-1^) = 1000 × (C_I_ × V_I_)/(C_W_ × W × t), where C_I_ is the concentration of PEG-4000 in the intestinal fluid (gL^-1^), V_I_ is the volume of intestinal fluid (mL), C_W_ is the concentration of PEG-4000 in experimental water (gL^-1^),W is the body weight of the fish (g), t is the duration of the experiment (h).

#### Molecular biology

The known sequences of the NKA-α gene of naked carp were compared with the corresponding genes of other species in GenBank, and highly conserved regions were selected for primer design (Table 1). The reference gene EF1α was used according to Yao et al.^3^. Previously published primers were used for SLC26A6 and SLC4A4 genes^6^. After extracting total RNA with Trizol (Invitrogen), the integrity of RNA was detected by 1% agarose gel electrophoresis, and the concentration and purity of total RNA were determined by a Bio Tek Epoch microplate spectrophotometer. The Rever Tra Ace-α (TOYOBO) kit was used to reverse transcribe mRNA to cDNA. Fluorescence quantitative PCR analysis was performed using a QuantStudio™ Real-Time PCR (Thermo life) with the SYBR Premix Ex TaqIII (TaKaRa) kit: total reaction volume of 10 μL, including 5 μL SYBR Premix Ex Taq, 2 μL upstream primers, 2 μL downstream Primers, and 1 μL cDNA template. The amplification procedure was as follows: 95 °C 30 s, 1 cycle; 95 °C 5 s, 60 °C 20 s, 40 cycles. Three replicates were included for each sample, with EF1α as the internal reference gene. The relative expression of each gene was calculated using the 2^−ΔΔCt^ method^41^. Melting curve analysis was performed following each reaction to confirm that there was only a single product and no primer-dimer artifacts. In addition, representative samples were electrophoresed to verify that only a single product was present. Negative control reactions were performed for representative samples using RNA that had not been reverse transcribed to control for the possible presence of genomic DNA contamination. No-template control reactions were also performed to verify the absence of contaminating DNA or primer-dimer amplification in the reactions.

**Table 1:** Nucleotide sequences of the primers used for amplification.

Primer	Primer sequences	References
EF1α F	5’-GTATTACCATTGACATTGC-3’	Yao et al.^3^
EF1α R	5’-CTGAGAAGTACCAGTGAT-3’	
NKA-α F	5’-CACGTGATGGACTCAACGCT -3’	–
NKA-α R	5’-TGGCTCCAATCCACAGGAGA-3’	
SLC4A4 F	5’-GCATTTATTCACTTTCGTCCAG-3’	Wang et al.^6^
SLC4A4 R	5’-AGATATAGTCCATCGCCTTCC-3’	
SLC26A6 F	5’-TGATTGGCAGTGTGACAGAG-3’	Wang et al.^6^
SLC26A6 R	5’-CAGTACAGTGGCAGCAGTAG-3’	

#### Single cell staining analysis

The naked carp mid-intestine was isolated and transferred to HBSS on ice. The mid-intestine was washed by HBSS (Corning, 21-022-CV) and transferred to pre-warmed digestion medium containing 0.2 mg·mL^−1^ Collagenase I (Gibco, 17100-017), 0.06 mg mL^−1^ Collagenase II (Gibco, 17101-015) and 0.2 mg mL^−1^ Collagenase IV (Gibco, 17104-019), which was shaken vigorously for 30 s and further incubated at 37 °C for about 30 min in incubator with gentle shaking every 5 min to release cells. Cells were then collected by centrifuging at 300 × g for 5 min, and resuspended in D-PBS (BBI, E607009-0500). Then taken an appropriate amount of single cell suspension and dropped it on poly-L-lysine-coated slides where the experimental area was drawn with a hydrophobic marker to allow the single cells to settle freely. When the cell sedimentation density was moderate, aspirated the excess cell suspension, slides were fixed with 4% paraformaldehyde fix solution (BBI, E672002-0500) for 10 min, and blocked with 3% BSA (Sigma, B2064) for 1 h, three washes in D-PBS. Subsequently, slides were incubated in NKA-α or SLC26A6 (antibody dilution ratio was 1:100) for overnight at 4 °C. The NKA-α antibody was a commercial polyclonal rabbit Na^+^/K^+^-ATPase α antibody (Santa Cruz Biotechnology, sc-28800). The SLC26A6 antibody was a commercial polyclonal rabbit SLC26A6 antibody (Abcam, ab-172684). After the incubation, three washes in D-PBS. The secondary antibodies consisted of Alexa flour 568 goat anti-rabbit IgG (Thermo Fisher Scientific, A11036) (antibody dilution ratio was 1:400). Slides were incubated in room temperature for 1 h, followed by three washes in D-PBS. Finally, incubate with Hochest for 30 min. Cells were then photographed with a fluorescence microscope. For every fish, positive protein expression was counted using at least three pictures. Image J was used to analyze the fluorescence intensity and record the positivity rate.

#### Statistical analysis

The data was expressed as mean ± standard error (SE). Two-way ANOVA and One-way ANOVA with LSD multiple comparison were employed to compare drinking rate, food intake and relative gene expression among different treatments and time courses respectively. Differences in single cell positive rate between 16:00 and 22:00 in L17 were evaluated by chi-square test. Assumptions for all parametric models (normality and equal residuals) were assessed via diagnostic plots. Means were considered significantly different when *P* < 0.05. All statistical analyses were conducted with SPSS 11. The bar charts were created using Origin 8.6.

## Supplementary Information

Supplementary Information.
