# Supplementary material for: Intestinal ion regulation exhibits a daily rhythm in Gymnocypris przewalskii exposed to high saline and alkaline water
Source: Sci Rep. 2022 Jan 17;12:807. doi: 10.1038/s41598-021-04472-5 (PMC8764090; doi:10.1038/s41598-021-04472-5)

Supplementary figures

Figure Legends

Fig.S1 The western blot analysis data for NKA-α and SLC26A6 (Western blot analysis for NKA-α and SLC26A6 revealed distinct bands of ~ 113 kDa and ~ 50 kDa respectively, the edges of some membrane were not shown because some blots were cut prior to hybridisation with different antibodies)

Figure S2. A schematic diagram of naked carp using self-feeder, including tank(1), feeder(2), photoelectric sensor(3), optical emitter(4), optical receiver(5), feeding ring(6), protector(balloon)(7)

FigureS1

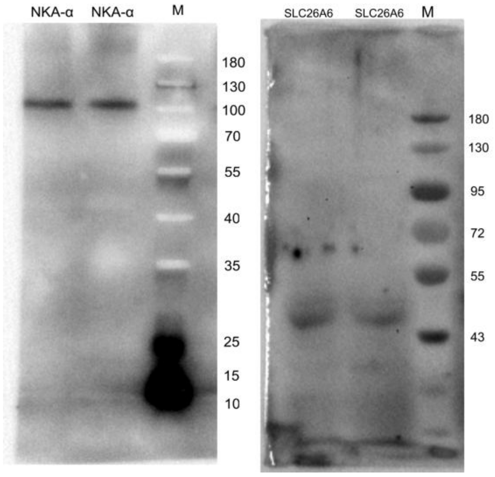

FigureS2

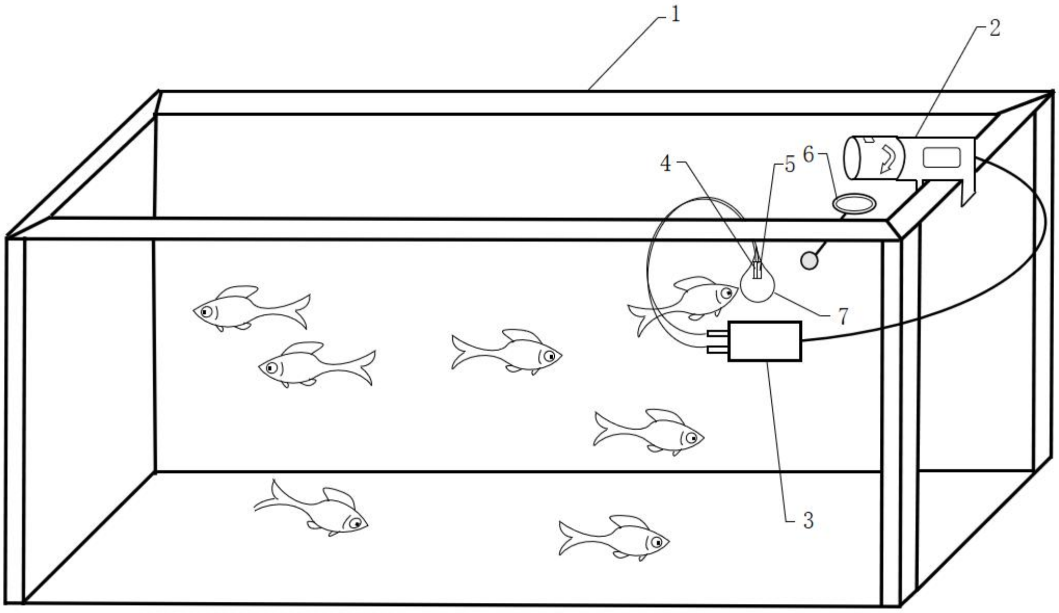

Supplement: Supplementary file 1 — Supplementary Information. [file 41598_2021_4472_MOESM1_ESM.pdf]
